# Publisher's Note

**DOI:** 10.1080/20008198.2018.1511120

**Published:** 2018-09-07

The following article was originally published in issue 9(1). It has now been added to issue 9(S2) (Special Issue: Children and natural disasters):

**Posttraumatic stress and depressive symptoms in children after the Wenchuan earthquake**

Jin Cheng^a,b^, YiMing Liang^a,b^, Lin Fu^a,b^ and ZhengKui Liu^a^

^a^CAS Key Laboratory of Mental Health, Institute of Psychology, Beijing, China; ^b^Department of Psychology, University of Chinese Academy of Sciences, Beijing, ChinaDOI:10.1080/20008198.2018.1472992

Special Issue URL: https://tandfonline.com/toc/zept20/9/sup2?nav=tocList

The following article was originally published in issue 9(1). It has now been added to issue 8(S7) (Special Issue: ESTSS Highlights 2017 – Child Maltreatment Across the Lifespan):

**Training the next generation of psychotraumatologists: Collaborative Network for Training and EXcellence in psychoTraumatology (CONTEXT)**

Frédérique Vallières^a^, Philip Hyland^a,b^, Jamie Murphy^c^, Maj Hansen^d^, Mark Shevlin^c^, Ask Elklit^d^, Ruth Ceannt^a^, Cherie Armour^c^, Nana Wiedemann^e^, Mette Munk^e^, Cecilie Dinesen^e^, Geraldine O’Hare^f^, Twylla Cunningham^f^, Ditte Askerod^g^, Pernille Spitz^g^, Noeline Blackwell^h^, Angela McCarthy^h^, Leonie O’Dowd^h^, Shirley Scott^h^, Tracey Reid^i^, Andreas Mokake^j^, Rory Halpin^j^, Camila Perera^a,e^, Christina Gleeson^c,j^, Rachel Frost^c,h^, Natalie Flanagan^d,j^, Kinan Aldamman^a,e^, Trina Tamrakar^d,i^, Maria Louison Vang^c,g^, Larissa Sherwood^a,i^, Áine Travers^d,f^, Ida Haahr-Pedersen^a,g^, Catherine Walshe^c,h^, Tracey McDonagh^d,f^ and Rikke Holm Bramsen^d^

^a^Centre for Global Health, School of Psychology, Trinity College Dublin, Dublin, Ireland; ^b^National College of Ireland, Dublin, Ireland; ^c^Department of Psychology, Psychology Research Institute, Ulster University, Northern Ireland; ^d^Department of Psychology, University of Southern Denmark, Odense, Denmark; ^e^International Federation of the Red Cross Centre for Psychosocial Support hosted by Danish Red Cross, Copenhagen, Denmark; ^f^Probation Board of Northern Ireland, Belfast, Northern Ireland; ^g^Danish Children’s Centres, Odense, Denmark; ^h^Dublin Rape Crisis Centre, Dublin, Ireland; ^i^Police Service of Northern Ireland, Belfast, Northern Ireland; ^j^Spirasi, Dublin, Ireland

DOI: 10.1080/20008198.2017.1421001

Special Issue URL: https://tandfonline.com/toc/zept20/8/sup7?nav=tocList

